# Prediction of Protein–ligand Interaction Based on Sequence Similarity and Ligand Structural Features

**DOI:** 10.3390/ijms21218152

**Published:** 2020-10-31

**Authors:** Dmitry Karasev, Boris Sobolev, Alexey Lagunin, Dmitry Filimonov, Vladimir Poroikov

**Affiliations:** 1 Department of Bioinformatics, Institute of Biomedical Chemistry, Moscow 119121, Russia; boris.sobolev@ibmc.msk.ru (B.S.); alexey.lagunin@ibmc.msk.ru (A.L.); dmitry.alekseevich.filimonov@gmail.com (D.F.); vladimir.poroikov@ibmc.msk.ru (V.P.); 2 Department of Bioinformatics, Russian National Research Medical University, Moscow 117997, Russia

**Keywords:** protein–ligand interactions, proteochemometrics, local sequence similarity, prediction of target proteins

## Abstract

Computationally predicting the interaction of proteins and ligands presents three main directions: the search of new target proteins for ligands, the search of new ligands for targets, and predicting the interaction of new proteins and new ligands. We proposed an approach providing the fuzzy classification of protein sequences based on the ligand structural features to analyze the latter most complicated case. We tested our approach on five protein groups, which represented promised targets for drug-like ligands and differed in functional peculiarities. The training sets were built with the original procedure overcoming the data ambiguity. Our study showed the effective prediction of new targets for ligands with an average accuracy of 0.96. The prediction of new ligands for targets displayed the average accuracy 0.95; accuracy estimates were close to our previous results, comparable in accuracy to those of other methods or exceeded them. Using the fuzzy coefficients reflecting the target-to-ligand specificity, we provided predicting interactions for new proteins and new ligands; the obtained accuracy values from 0.89 to 0.99 were acceptable for such a sophisticated task. The protein kinase family case demonstrated the ability to account for subtle features of proteins and ligands required for the specificity of protein–ligand interaction.

## 1. Introduction

Discovery of the biologically active substances and their target proteins is a crucial stage in drug development. Computational prediction of the interaction between the proteins and drug-like ligands significantly decreases the time consumption and costs required for experimental studies.

The approach known as SAR (structure–activity relationship) allows for prediction of the biological activities of a compound from its structural features [1,2]. The SAR methods enable prediction of a particular activity for a new compound if there is a training set of compounds tested on the studied activity. In the case of proteins with unknown ligands, the SAR application for prediction of targets is impossible. Another way is modeling the 3D structure of the protein–ligand complex. The latter needs the solved 3D protein structure, which is not always available. Furthermore, it requires significant computational resources, especially at a screening of large chemical libraries.

Overcoming the mentioned limitations, the researchers investigate the combined space of protein and ligands. It allows for building large-scale predictive models [3]. Such an approach is known as “proteochemometrics” [4]. The predictive programs use various classifier techniques based on support vector machines (SVMs), artificial neural networks (ANNs), regression methods, etc. As the protein–ligand interaction is a complex process, the predictive model depends on the peculiarities of studied proteins and small-molecule ligands. Generally, the efficiency of various predictive methods depends on the way to describe the proteins and compounds [5]. The recent publications showed the broad applicability of this approach in predicting the promising drug targets [6,7,8,9,10,11,12,13,14,15,16,17,18,19,20,21,22].

Small-molecule ligands’ description uses the well-known techniques developed for SAR investigations [23]. Protein representation includes diverse techniques. Usually, they summarize the features describing the entire amino acid sequence [5]. The normalized scores of Smith–Waterman pairwise alignment are the typical characteristics [7]. Several approaches derive the sequence descriptors from the distribution of residues, correlation of physicochemical properties in residue pairs, etc. [10,22].

Generalized estimates do not account for the impact of individual amino acid residues. However, the experimental studies show examples when the individual amino acid residues crucially influence the protein affinity to a ligand [24,25]. In some cases, the residues distant from the binding site can significantly affect protein–ligand interaction [26]. 

Several approaches provide predictive models for the restricted set of target proteins and multiple ligands. In extreme cases, mutant variants of the same display various affinities to the compounds [9,13,17,22]. Such a predictive model requires the correct multiple sequence alignment [10,27]. The high similarity of studied proteins can provide an accurate matching of aligned residues. These methods are less suitable for studying the protein family, displaying a weak correlation between homology and ligand affinity like protein kinases.

We suggest the method that predicts protein–ligand interactions from the amino acid sequences classified by the protein affinities to ligands. Description of amino acid sequences calculated from a segment-to-segment comparison of the test and training sequences allows avoiding problems with the incorrect matching of aligned residues [28]. The earlier version of our method evaluated the possible interaction of protein–ligand pairs by comparing a test sequence with the training proteins partitioned into classes of ligand interactors and non-interactors [29]. Each training sequence got two binary coefficients that defined interaction and non-interaction with each ligand within a training set. Superimposing sequence segments of the test sequence and a training one, the program calculated the positional similarity scores. The latter were weighted by the belonging coefficients and integrated on the entire training sets to obtain the estimate of protein–ligand interaction. Thus, we provided the prediction of new target proteins for ligands with the known sets of targets (target spectra).

Therefore, the binary coefficient 1 and 0 were used to define the protein belonging to one of two clear classes presenting the targets interacting and not interacting with a given ligand, respectively. In this study, we also applied the fuzzy classification of proteins. All proteins from a training set were referred to the class of interacting targets with different belonging coefficients. Belonging to the class of non-interacting proteins is defined in the same manner. These “fuzzy” coefficients adopted values in the range from 0.0 to 1.0. They were calculated from ligands’ structural features with the PASS (prediction of activity spectra for substances) method, successfully used in many SAR studies [30,31]. Our results confirmed the efficacy of a suggested approach for estimating the possible interaction if both the target and ligand undescribed. We showed the applicability of the suggested method for protein families differed by the included proteins’ diversity.

## 2. Results and Discussion

We tested our program on five protein groups: G protein-coupled receptors (GPCRs), protein kinases, ligand-gated ion channels, voltage-gated ion channels, and nuclear receptors. The characteristics of training sets collected according to the below-described procedure (see Section 3.1 and Section 3.2) are present in Table 1. The entire datasets are available online (http://www.way2drug.com/proteochem_dataset.php).

According to protein groups, interaction parameters (IC50, Kd, and Ki), and cutoff values (1 and 10 µmol), the collected data formed several datasets.

As described below, each protein–ligand pair in a training set got the interaction index equal to 1 (interaction) or 0 (no interaction). Thus, we composed up to six sets for each protein group (Table 1).

Commonly, three predictive tasks are considered in predicting protein–ligand interaction:

(a) A new ligand for a target with a known ligand spectrum based on the chemical structure comparisons;
(b) A new target for a ligand with a known target spectrum based on the protein sequence comparisons;
(c) A new protein–ligand pair if both objects were uncharacterized. It includes a comparison of ligand structures as well as a comparison of protein sequences.

**Figure 1:**
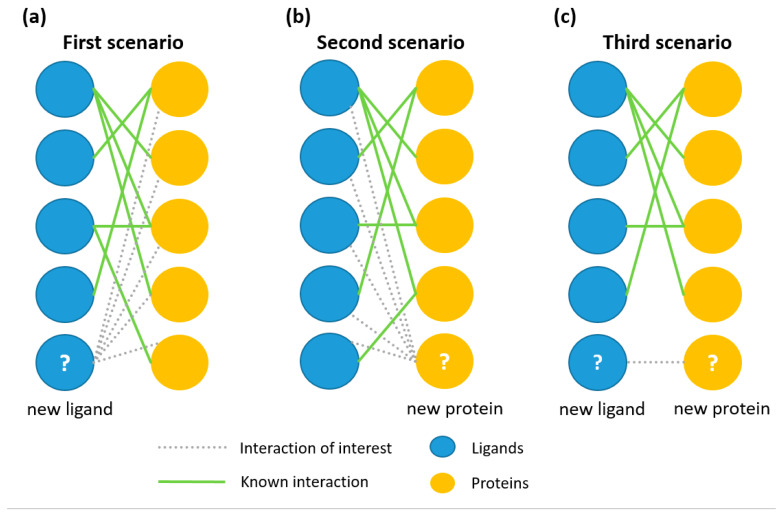
Scenarios simulating prediction of new ligands for known proteins (**a**), new targets for known ligands (**b**), and the interaction between new ligands and new targets (**c**).

To simulate all situations and test our approach, we implemented three scenarios (Figure 1).

In all cases, the prediction accuracy was evaluated with ROC (receiver operating characteristic) analysis based on the leave-one-out cross-validation procedure. This technique provides building the dependence curve of true positive vs. false positive rates at different cutoffs of accuracy scores. The area under curve (AUC) is commonly used as accuracy estimate [32].

In the first scenario, each ligand is alternately removed from a training set and handled as a test one. The PASS-program predicted this compound’s affinity to all training targets, resulting in probability estimates *P_a_* and *P_i_* (see Section 3.3).

After performing this procedure for each ligand, the probability estimates were used for ROC analysis [30]. As a result, the AUC values for all training sets varied from 0.80 to 0.99 with 0.96, on average (Table 2). Interestingly, the ligand estimates on six protein kinase datasets showed the most AUC variability, comparing the other target groups. It could be explained by the close structural similarity of ligand-binding pockets in proteins of this superfamily [33]. However, the different ligands display the paradoxical selectivity to different kinases subclasses and proteins. Thus, such specificity seems to be related with subtle features responsible for target specificity and sufficiently large number of compound structures is required to recognize them. Indeed, the lowest numbers of ligands in the training sets, 111 (Kd, 1 µmol) and 120 (Kd, 10 µmol), corresponded to the lowest AUC values 0.81 and 0.80 (see Table 1 and Table 2), respectively. The high target prediction accuracy allowed us to accept the obtained probability estimates as belonging coefficients in the third scenario (see Section 3.4).

In the second scenario, we applied our earlier developed method SPrOS [29], which predicts new targets for ligands with known target spectra. In each step, the next protein was considered as unknown to interact with any target.

The training sets’ proteins were partitioned into ligand-class specificity with the binary coefficient, as described in Section 3.4. The prediction of protein affinity to all compounds was performed (Table 3). We evaluated the relation of protein–ligand affinity and protein sequence ordering by using two frames (length of compared sequence fragments, see Section 3.5) values of 7 and 30 residues. These frame values were selected in our preliminary studies as representing the close and distant inter-residue interactions [29]. The obtained estimates were used for further ROC analysis.

Testing the GPCRs at all parameters (parameter type, cutoff, and frame) showed the high ligand prediction accuracy with the average AUC value of 0.95 with small variation. Thus, our approach revealed the stable sequence features required for the ligand specificity.

Predicting the ligand-specificity of protein kinases resulted in the less accurate estimates, displaying the average AUC value of 0.84 with significant variation from 0.69 to 0.94. Using the frame of 30 residues somewhat increased the predictive power compared with the seven-residue frame. It seems to relate to the more distant residue interacting. The stricter cutoff of 1 µmol conditioned the significantly more accurate prediction, which is consistent with results obtained for the kinases’ ligand prediction.

The three remaining protein groups presented a relatively small number of sequences and ligands. However, predicting the ligands for these groups displayed high efficiency (Table 3). The average AUC values for nuclear receptors, ligand-gated ion channels, and voltage-gated ion channels were 0.99, 0.98, and 0.94, with small differences associated with dataset parameters.

In general, the results obtained in the second scenario are close to the results published in our previous study, which used the same methods and provided prediction accuracy comparable or exceeding those of other methods with the “Gold Standard” set [7].

The third scenario differed from the second one by using fuzzy interaction coefficients besides binary coefficients. The fuzzy coefficient presented the probabilities of interaction (*P_a_*) and non-interaction (*P_i_*), as calculated in the first scenario. This technique (see Section 3.4) should allow the prediction of unavailable data for both the protein and ligand. The ROC analysis of prediction results uses the binary coefficients.

The obtained AUC values were expectedly lower than those calculated in the second scenario (Table 3). However, the values calculated for GPCRs were high enough, reaching the maximal value of 0.92 (Ki, cutoff = 10 µmol, frame = 30). The kinase-ligand interaction was predicted with the more variable accuracy from rather small 0.64 (Kd, cutoff = 1.0 µmol, frame = 7) to quite acceptable 0.89 (Ki, cutoff = 1 µmol, frame = 30). In two mentioned larger protein groups, the prediction estimates tended to be higher at the 30-residue frame. The kinases’ group demonstrated a higher accuracy at the 1.0 µmol cutoff. This evidence looks interesting, but its significance is not quite clear now. The accuracy estimates for nuclear receptors and ion channel groups obtained in the second and third scenarios were very close and high at all used parameters.

**Figure 2:**
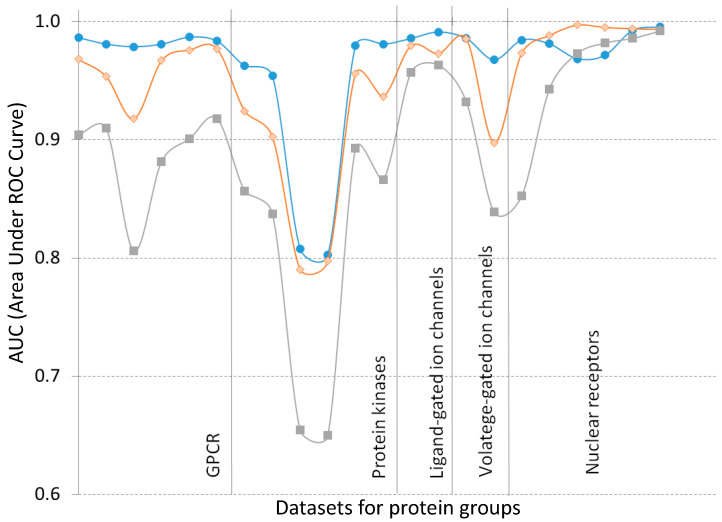
The accuracy prediction obtained for datasets related to five protein–ligand groups. Each point corresponds to the AUC value from Table 2 and Table 3. The blue, orange, and gray curves are related to the first, second, and third scenario, respectively. Each AUC value equals to maximum of ones calculated at frames of 7 and 30.

Resuming the results, we could have presented the dataset on each protein–ligand group. It allowed obtaining enough high accuracy in all three scenarios, including the case of both the new interactors (Figure 2). All three curves are similar in their behavior, especially in the datasets of protein kinase. This protein family demonstrated a lower accuracy in all scenarios. It corresponds to the weak relation of phylogeny and ligand specificity found for this protein family. However, the two kinase sets allowed predicting with quite the acceptable accuracy even in the third scenario. The similar shapes of curves obtained in the first and second scenarios demonstrated the correspondence between protein and ligand features responsible for their interaction. It spoke in favor of applying the third scenario technique if the protein and target are new.

Our previous study showed the high efficiency of the proposed approach in predicting protein–ligand interaction [29]. The binary interaction coefficients based on experimental data were applied in that work, allowing prediction of new ligands for targets with known ligand spectra. We investigated the various situations observed in proteochemometrics, including the cases of both new target and ligand. In the latter task, the probability coefficients calculated from ligand structure similarity defined the fuzzy classes of ligand specificity for target proteins. It required more careful preparation of the training data. For comparing our and other methods’ predictive power, we used in that study the well-known dataset “Gold Standard” as benchmark data [7]. To account for the detailed characteristics of experimental source data, we collected the own datasets, defined by interaction index type and affinity cutoff, and directly extracted from the ChEMBL database with a robust filtration procedure. It allows us to test our method for various proteochemometrics tasks rigorously.

## 3. Material and Methods

### 3.1. Data Preparing

The training set should contain enough reliable data confirmed at the molecular level, requiring careful processing of the information from available resources. The selected data should present the ligands’ chemical structures, target proteins’ sequences, and the affinity values for each protein–ligand pairs.

We used the local version of the ChEMBL 25 database, which contains detailed experimentally obtained characteristics of interacting the small molecules and target proteins, including the ligands’ chemical structures, proteins’ peculiarities, and study condition [34]. 

To select reliable and suitable data, we formed the MySQL queries with the following limitations:

- Direct testing of the protein–ligand binding;
- The accurate definition of a protein (neither a homolog nor a protein family);
- Testing the isolated protein (it is not in a complex or cellular fraction, etc.);
- IC50, Ki, Kd as interaction parameters;
- Excluding the mutant proteins.

The ligands’ structures were normalized with the RDkit package (https://www.rdkit.org) at default parameters. According to the limitations of the PASS program [30], the compounds with charged atoms were excluded as well as molecules whose mass was greater than 1250 Da. The compounds with the same MNA descriptor set and molecular weight were considered as the same compound [31].

By extracting the most recent data from the ChEMBL database with strong filters, we obtained reliable and representative data sets for protein groups known as drug targets: protein kinases, nuclear receptors, G protein-coupled receptors (GPCR), voltage- and ligand-gated ion channels.

### 3.2. Activity Definition

The data retrieved for the same interacting pair often presented the results obtained by different research groups at different experimental conditions. Therefore, we developed an algorithm that provided the integration of such data. According to protein–ligand interaction parameters, we divided the dataset composed for each protein group into three subsets: IC50, Ki и Kd, if enough data were available. These parameters adopted precise (such as 0.1 µmol) or interval values (such as >0.1, <50.0 µmol, etc.). In all three cases, we established two cutoffs, 1.0 and 10.0 µmol, and considered the protein and ligand as interacting if the parameter did not exceed the cutoff. As a result, each protein–ligand pair got the binary index designating the interaction (1) or non-interaction (0). As the calculation of this interaction index affected the dataset size, three mentioned subsets were divided into two parts, resulting in up to six subsets for each protein group if data were available.

We calculated the interaction index values according to the following rules:

- In the presence of both fixed and interval values, only fixed parameters were accounted for if even interval values contradicted fixed ones. The interaction index adopted a value of 1 if the data median did not exceed the cutoff and 0 otherwise;
- In the absence of fixed values, the protein–ligand pair was not considered if interval values put in non-intersected areas (e.g., <100 and >5000) or the parameter values were at the interval limited at two ends (e.g., >100 and <5000);
- In the case of a few intervals limited below (e.g., >100, >1000, >5000), the index adopted 0 if the maximal edge value was higher than a given cutoff; otherwise, the protein–ligand pair was excluded;
- In the case of a few intervals limited above (e.g., <100, <1000, <5000), the index adopted a value of 1 if the minimal extreme value was less than the cutoff; otherwise, the protein–ligand pair was excluded.

The datasets are available online at http://www.way2drug.com/proteochem_dataset.php.

Each target protein in the training set should have at least three established ligands, providing an adequate PASS program prediction. Each ligand should have at least three established targets, providing the correct ROC analysis.

### 3.3. Prediction of the Target Proteins from Ligand Structural Features

The PASS method was developed for predicting the biological activities of drug-like organic compounds [30]. This method uses the original descriptors of the multilevel neighborhoods of atoms (MNAs), which reflect the local features of ligand–target interaction. It is based on a naive Bayesian classifier [30]. For each target–ligand pair, the PASS program outputs *P_a_* and *P_i_* scores, which are probabilities of interaction and non-interaction between target protein and ligand, respectively.

### 3.4. Retrieving the Fuzzy Coefficients for Target–Ligand Pairs

In this study, we simulated the situation with uncharacterized ligands and sequences using the belonging fuzzy coefficients calculated by the PASS program. Comparing the results obtained with fuzzy and binary coefficients showed the usefulness of the suggested approach.

Using the PASS program, we obtain the fuzzy classes, including all training set ligands, introducing Pa and Pi as coefficients belonging to the class of ligands of a particular target protein. It allows predicting affinity for the protein–ligand pairs with non-defined interaction spectra for both the protein and compound. Most training data have no assignment because of the absence of experimental results, and they are usually considered conditionally negative examples. However, some of them can present positive but non-detected examples. Using the weight coefficient based on structural similarity enables accounting for hidden positive examples.

### 3.5. Prediction of Protein–Ligand Interactions

Our approach uses the positional scores calculated by segment comparison between the test sequence and all training sequences [29]. The segment-to-segment comparison of the test and training sequence (*K*) results in the segment scores *R* calculated as follows:

(1) $$R_{ih}=\sum_{j=i}^{j+F-1}sim\left(q_{j,}k_{j+h}\right)$$

where *F* is the segment length, *i* is the position of a sequence *Q*, *h* is a shift between sequences, *sim* (*q_n_, k_m_*) is a similarity of matched residues from *Q* and *K* sequences accordingly to a chosen metrics (residue identity in this study).

Finally, the score for each position *p* equals the maximum of *R* values obtained for all segments containing *p* at all shifts.

(2) $$S_{pk}=\underset{h,i}{\max}R_{ih},p-F<i\leq p$$

The positional scores obtained with all training sequences are integrated to estimate the possible protein–ligand interaction.

Commonly, proteochemometrics studies aim at the solution of two tasks. One of them is an estimation of belonging the protein to the class of ligand targets. Another one is an estimation of belonging the compound to the class of protein ligands. The incompleteness of experimental data significantly decreases the number of bound protein–ligand pairs included in the training set. We suggest overcoming this limitation by introducing weight coefficients based on compounds’ structural peculiarities (see Section 3.3). Each *k^th^* training sequence gets two coefficients *a^k^* and *b^k^*, which, respectively, weight the protein ability and inability to interact with a given compound *C*. In other words, these values define a priori belonging a protein to the classes of ligand interactors and non-interactors.

Estimating the potential protein–ligand affinity uses the naïve Bayes according to the technique described earlier for chemometrics methods [30].

During the prediction procedure, the test sequence *Q* should be compared with *N* training sequences. The integrated score for each position of *Q* is calculated as follows:

(3) $$t_{p}=\frac{\sum_{k=1}^{N}S_{pk}\times \left[a_{k}(C)-b_{k}(C)\right]}{\sum_{k=1}^{N}S_{\begin{array}{l} pk \end{array}}\times \left[a_{k}(C)+b_{k}(C)\right]}$$

where *S_pk_* is a score for the position *p* obtained by comparison with the *k^th^* sequence.

The *t_p_* values are average on all *m* positions of the test sequence

(4) $$t=\sin\left[\frac{1}{m}\sum_{p=1}^{m}\arcsin(t_{p})\right]$$

The a priori value *t_0_* presents weights of ability and inability to interact with a ligand without accounting for sequences similarity:

(5) $$t_{0}=\frac{\sum_{k=1}^{N}\left[a_{k}(C\right)-b_{k}(C)]}{\sum_{k=1}^{N}\left[a_{k}(C\right)+b_{k}(C)]}$$

Finally, the possible interaction of a test protein with ligand *C* is estimated as follows:

(6) $$B(C)=\frac{t-t_{0}}{1-tt_{0}}$$

The estimate *B(C)* varies from −1 (no interaction) to +1 (interaction). The null value shows the undetermined result.

Our previous study used binary coefficients of protein–ligand interaction and non-interaction to predict new target proteins for ligands with known target spectra [29]. This study applies the PASS program to obtain the fuzzy coefficients based on ligands’ structure similarity. Thus, the ligand with experimentally non-established targets can be accounted for in the prognosis of protein–ligand interactions. In other words, we can estimate the possible interaction of a new target protein and new ligand. Briefly, to predict a pair of the new target and new ligand, the PASS program should evaluate test ligand’s potential interaction with training proteins based on their ligand structures. Probabilities *P_a_* and *P_i_*, reflecting the *k^th^* protein’s affinity to a test ligand, substitute values *a_k_* and *b_k_* into Equations (3) and (5).

## 4. Conclusions

Our study’s principal aim was to develop the method for predicting the protein–ligand interaction based on the protein sequences and ligand structures. Defining targets with enough narrow sets of experimentally detected or predicted ligands as well as ligands with narrow set of targets is promised approach to obtain selective drug candidates.

To correctly test our approach, we collected the datasets with various parameters of interaction. The applied procedure overcame difficulties caused by the ambiguity of initial information. The collected data are available online (http://www.way2drug.com/proteochem_dataset.php).

We implemented fuzzy coefficients weighting the interactions of target proteins and small molecule compounds to solve this task. The PASS software provided the reliable probabilistic estimates of ligand affinity to targets. Applying these values as weight coefficients, we modified the earlier prediction method using the binary prediction coefficients. Comparing the results obtained with the previous and new program versions showed the expected accuracy decrease with fuzzy coefficients. However, the estimates calculated in the second case were quite acceptable with the most dataset. Generally, we defined the more suitable conditions of each protein group’s predictive procedure by testing our method with various parameters and the best results for GPCR, protein kinases, ligand-gated ion channels, voltage-gated ion channels and nuclear receptors were 0.92, 0.89, 0.96, 0.93, and 0.99, respectively (Table 3). Indeed, the more precise definition of ligand specificity with binary coefficients should condition the more prediction accuracy. Testing with fuzzy weights showed the suggested approach’s applicability to predict the pairs of both new interactors. 

Protein kinases displayed the lowest estimates. These results seemed to relate with smaller structural differences in these target proteins and ligands, causing the less effective recognition of interaction specificity. High accuracy was detected with binary and fuzzy coefficients for a few datasets (Figure 2). 

The correspondence between the protein and ligand features shown in our study spoke in favor of cooperative using protein and ligand description, as simulated in the third scenario.

Our approach is suitable for the broad applicability domain in proteochemometrics with different degree of correlation between the phylogenic relations and ligand specificity. In case of protein kinases demonstrating the weak dependence of that kind, our method provided prediction of ligand specificity with acceptable accuracy. It suggests that our method is suitable for not yet considered protein targets and their ligands, given the enough representative data sets.

## Figures and Tables

**Table 1:** Characteristics of the training sets representing the protein–ligand interactions.

Protein Group	Parameter	Cutoff µmol	Number of Target Proteins	Number of Ligands
GPCR *	IC50	1	126	546
		10	130	839
	Kd	1	30	8
		10	32	15
	Ki	1	110	4754
		10	112	6411
Protein kinases	IC50	1	200	3014
		10	215	3883
	Kd	1	233	111
		10	307	120
	Ki	1	72	277
		10	77	339
Ion channel (ligand-gated)	Ki	1	16	15
		10	16	20
Ion channel (voltage-gated)	IC50	1	29	75
		10	35	163
Nuclear receptors	IC50	1	26	121
		10	28	340
	Kd	1	13	15
		10	13	19
	Ki	1	22	55
		10	23	89

* G protein-coupled receptors.

**Table 2:** Predictive accuracy for ligands’ affinities to proteins (first scenario).

Protein Group	Parameter	Cutoff µmol	AUC *
GPCR	IC50	1	0.986
		10	0.981
	Kd	1	0.979
		10	0.981
	Ki	1	0.987
		10	0.984
Protein kinases	IC50	1	0.963
		10	0.954
	Kd	1	0.808
		10	0.803
	Ki	1	0.980
		10	0.981
Ion channel (ligand-gated)	Ki	1	0.986
		10	0.991
Ion channel (voltage-gated)	IC50	1	0.986
		10	0.968
Nuclear receptors	IC50	1	0.984
		10	0.981
	Kd	1	0.969
		10	0.972
	Ki	1	0.993
		10	0.996

* Area Under ROC Curve.

**Table 3:** Predictive accuracy (AUC) for target affinities to ligands (second scenario) and both uncharacterized interactors (third scenario).

Protein Group	Parameter	Cutoff µmol	Second Scenario		Third Scenario	
			Frame = 7	Frame = 30	Frame = 7	Frame = 30
GPCR	IC50	1	0.959	0.968	0.885	0.904
		10	0.944	0.953	0.890	0.910
	Kd	1	0.918	0.875	0.806	0.805
		10	0.967	0.962	0.874	0.882
	Ki	1	0.963	0.976	0.881	0.901
		10	0.966	0.977	0.899	0.918
Protein kinases	IC50	1	0.896	0.924	0.824	0.857
		10	0.866	0.902	0.769	0.838
	Kd	1	0.686	0.790	0.642	0.655
		10	0.710	0.797	0.647	0.650
	Ki	1	0.930	0.956	0.869	0.893
		10	0.907	0.937	0.866	0.856
Ion channel (ligand-gated)	Ki	1	0.979	0.979	0.948	0.957
		10	0.970	0.973	0.958	0.963
Ion channel (voltage-gated)	IC50	1	0.985	0.985	0.913	0.932
		10	0.886	0.898	0.787	0.839
Nuclear receptors	IC50	1	0.966	0.973	0.817	0.852
		10	0.984	0.988	0.924	0.943
	Kd	1	0.995	0.995	0.973	0.961
		10	0.995	0.995	0.982	0.972
	Ki	1	0.994	0.988	0.986	0.976
		10	0.993	0.987	0.992	0.989
